# Biodetoxification and Protective Properties of Probiotics

**DOI:** 10.3390/microorganisms10071278

**Published:** 2022-06-23

**Authors:** Oana Lelia Pop, Ramona Suharoschi, Rosita Gabbianelli

**Affiliations:** 1 Department of Food Science, University of Agricultural Sciences and Veterinary Medicine, 400372 Cluj-Napoca, Romania; ramona.suharoschi@usamvcluj.ro; 2 Molecular Nutrition and Proteomics Laboratory, Institute of Life Sciences, University of Agricultural Sciences and Veterinary Medicine, 400372 Cluj-Napoca, Romania; 3 Unit of Molecular Biology and Nutrigenomics, Via Gentile III da Varano, 62032 Camerino, MC, Italy

**Keywords:** probiotics, food contaminants, detoxification, antimutagen, anticarcinogen

## Abstract

Probiotic consumption is recognized as being generally safe and correlates with multiple and valuable health benefits. However, the mechanism by which it helps detoxify the body and its anti-carcinogenic and antimutagenic potential is less discussed. A widely known fact is that globalization and mass food production/cultivation make it impossible to keep all possible risks under control. Scientists associate the multitude of diseases in the days when we live with these risks that threaten the population’s safety in terms of food. This review aims to explore whether the use of probiotics may be a safe, economically viable, and versatile tool in biodetoxification despite the numerous risks associated with food and the limited possibility to evaluate the contaminants. Based on scientific data, this paper focuses on the aspects mentioned above and demonstrates the probiotics’ possible risks, as well as their anti-carcinogenic and antimutagenic potential. After reviewing the probiotic capacity to react with pathogens, fungi infection, mycotoxins, acrylamide toxicity, benzopyrene, and heavy metals, we can conclude that the specific probiotic strain and probiotic combinations bring significant health outcomes. Furthermore, the biodetoxification maximization process can be performed using probiotic-bioactive compound association.

## 1. Introduction

Food is vital for human health, delivering energy, and nutrients, and plays crucial roles in the human body, tissues, growth and development of organs, normal function, and metabolism [1,2]. Besides nutrients, food could have traces of different toxins (as naturally occurring or by-products during food processing or storage), usually at non-detectable levels and below unobserved adverse effect levels. Food toxins, including fungi (yeasts and molds), industrial waste contaminants—heavy metals (arsenic (As), cadmium (Cd), mercury (Hg), and lead (Pb)), acrylamide, and benzopyrene, increase the risk of dysbiosis, mutagenesis, and carcinogenesis [3,4,5]. Food and feed contamination are almost impossible to entirely avoid. Instead, the adoption of various measures to detoxify contaminated food and feed is more feasible and necessary. Several techniques (physical, chemical, and biological) have been studied to detoxify and mitigate hazards affecting the population’s health and significantly diminish the economic damage caused by these toxins in food and feed. These methods act by destroying or modifying the toxin’s molecular structure, resulting in the toxin’s low accessibility to the digestive system [1,3,4,6].

The toxic chemical biodetoxification could be associated with the gut microbiota, which is essential for maintaining intestinal integrity in the longer term. Overall, the gut tract’s microbiota might also be crucial for in vivo biodetoxification. In the past decades, probiotics have raised interest due to their comprehensive properties, not only in the digestive system but also in vivo biodetoxification [7,8,9]. This review aims to demonstrate the current evidence-based supporting probiotics biodetoxification and protective activity. The main question that leads this study reveals the specific mechanisms involved in the detoxification process, and the conditions that influence these mechanisms. Since most toxic compounds induce carcino- and mutagenesis, particular focus is given to probiotics’ anti-carcinogenic and antimutagenic action.

## 2. Probiotics in Human Health and Microbiota Modulation

The term “probiotic” means “for life” and has its origins in ancient Greece. Probiotics have recently been classified into two groups, including nutribiotics (food) and pharmabiotics (drug) [10]. Currently, ethnic and industrial probiotic food products are excellent modalities for increasing regular probiotic ingestion [11]. In the summer of 2014, a paper discussed the International Scientific Association for Probiotics and Prebiotics (ISAPP) gathering from 2013, on the topic of probiotic term appropriate usage. They decided to retain the probiotics definition given by FAO/WHO “live microorganisms that, when administered in adequate amounts, confer a health benefit on the host” [12]. This definition is broadly accepted in the scientific world. In addition, scientists sustain defining the term postbiotic [13,14], where viability and colonization are not required for the exercitation of health benefits, for some genera [14,15]. 

Most probiotic foods contain microorganisms belonging to *Lactobacillus*, *Bifidobacterium*, *Streptococcus*, *Enterococcus*, *Escherichia*, *Bacillus*, and yeast genera, mostly the *Saccharomyces* species [6,16].

It has been reported that several chronic and degenerative diseases have direct implications for gut microbiota dysbiosis. In some cases, this dysbiosis may trigger these diseases and affect mood and behavior (depression, anxiety, and stress) [11,17]. 

Certain conditions during the perinatal period can impair the normal development of the gut microbiota. Promoting breastfeeding, reducing hospital stay, not consuming unnecessary antibiotics, and using some pre/probiotic supplementation could prevent the alteration or dysbiosis of the gut microbiota [1,18]. In microbial equilibrium, probiotics play an essential role, as well as enhance vaginal and urinary health, skin conditions, prevention of allergies, colds, bone health, lactose intolerance, diarrhea, GI (gastrointestinal) inflammation, and allergies [1,9,16]. 

Recent studies have shown that, even if probiotic mechanisms of action are not fully understood, probiotic multiple strain mixtures are more effective, and new strains with specific actions need to be isolated [2,3,19]. Although close to exhaustive investigations of the human microbiota composition have been conducted, their functions and functionalities still need to be elucidated based on solid evidence for understanding their simultaneous involvement in diseases and treatments’ etiology [1]. Therefore, individuals’ gut microbiota composition is an essential tool for determining the probiotic treatment strategy. 

Probiotic metabolites bring health benefits to the human body. Bioactive compounds are formed by probiotics, including bacteriocins, enzymes, amino acids, peptides, short-chain fatty acids, and anti-inflammatory and immune-modulating agents [17,19,20]. Concretely, the gut epithelium is protected, inflammation is reduced, and the immune profile is modulated. The prevention, treatment, and alleviation of a broad range of diseases can enforce discussions on recent microbial-based cure discoveries. However, while modern probiotic therapies have a promising perspective, regulations are required for their authentic development [21].

The scientific literature sustains the use of probiotics in a broad range of pathologies (enteric-related ones being most studied) with positive results, in several ways of action (decreasing intestinal pH, lowering colonization and multiplication of pathogens, metabolites, boosting the host immune response, bind toxins) [10,19,22,23,24,25,26,27,28,29,30]. The main pathway by which probiotics influence human health is their ability to protect and ensure good function of the gut epithelium by (i) direct influence on the epithelium—mucin expression and secretion by goblet cells and increase β-defensin secretion by the epithelial cells; (ii) increase mucosal immunity—increasing IgA-producing cells; and (iii) reduce pathogens numbers and/or their gene expression [15,30]. 

In their study, Barouei et al. show how mucin secretion, is sustained by down-regulation of plasma IFN-γ and haptoglobin in the presence of *B. animalis* subsp. lactis BB-12 and *Propionibacterium jensenii* 702 in concentration of 3 × 10^9^ and 8.0 × 10^8^ CFU/mL respectively [31]. Yang et al. 2012 demonstrate the protective effect of yogurt probiotics (*L. acidophilus*, *B. lactis*, *L. bulgaricus*, and *Streptococcus thermophilus*) in *Helicobacter pylori* infection by restoring affected *Bifidobacterium* in the gut microflora and by increasing serum IgA titer (low in *H. pillory* infection) [32].

In most cases, the protective effect of probiotics, in various pathologies, is based on multiple ways of action. 

## 3. Probiotic Safety Issues

**Figure 1:**
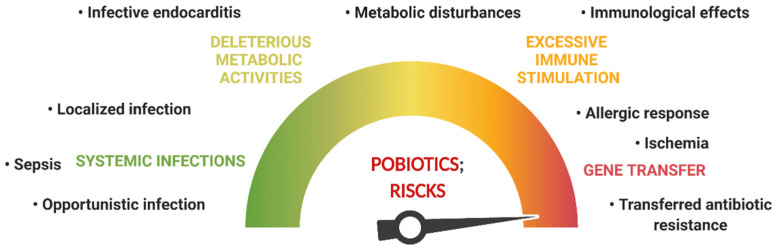
Possible risks caused by probiotics.

It is widely accepted that the most used probiotic strains are safe for usage [16,21]. These strains received the status “qualified presumption of safety.” The safety assessment should include the type of microorganism being used, the method of administration, the exposure levels, and the consumer’s health status. While probiotics are commonly acknowledged as safe for healthy subjects, few pieces of evidence emphasize the contrary for certain groups with unique risks [33]. Nevertheless, the potential benefits of probiotics compensate for the potential risks when considering the long-term. Probiotic species may have a natural origin or could be genetically engineered (tailored probiotics) for a specific effect (i.e., expressing a specific protein, biomaterial delivery, annihilating infectious pathogens to combat infectious, and metabolic diseases), so their impact on human health may differ, or their mechanisms of action may vary [34]. The FAO research group reports that probiotic action in patients with special medical status could be associated with four specific forms of side effects and risks (Figure 1): “(1) systemic infections; (2) deleterious metabolic activities; (3) excessive immune stimulation in susceptible individuals; and (4) gene transfer” [35].

Probiotic functional foods or supplements may contain a single or mix of bacteria species on the market. Several products containing probiotics, including milk, infant formula, cheese, drinks, and dietary supplements, are marketed in classical or novel procedures worldwide. This aspect results in the large ingestion of probiotic cells and significant interaction with various gut microbes at high densities. Thus, any gene resistant to antibiotics carried by probiotic cells may be relocated to the gut microorganisms, including pathogens [36]. Therefore, we should consider the risk of ingesting antibiotic resistance genes or antibiotic-resistant bacteria.

The genes in *Bacillus* probiotics indicate a potential health risk due to the production of their toxins, and they harbor various antimicrobial resistance genes [37].

Probiotics could have adverse effects if used inappropriately or if they do not meet the required standards [38]. Indeed, their application in preventing, ameliorating, or treating some diseases is essential, but knowing and facing the other side is crucial. In specific cases, probiotics and probiotic mixt administration in high-risk populations may result in health complications [39]. Therefore, we conclude that probiotic supplements can be effective in different age groups of consumers and should be wisely selected. Furthermore, it is prudent to take precautions when administering probiotics.

## 4. Food Contaminants and Their Impact on Human Health

In specific cases, food can be hazardous to one’s health, causing disease and death. Approximately two million people die annually, including children, due to contaminated foods full of harmful chemicals (heavy metals, acrylamide, polycyclic aromatic hydrocarbon—benzopyrene) or biological (microorganisms, pathogens, fungi—molds, and yeasts) compounds [40,41].

**Figure 2:**
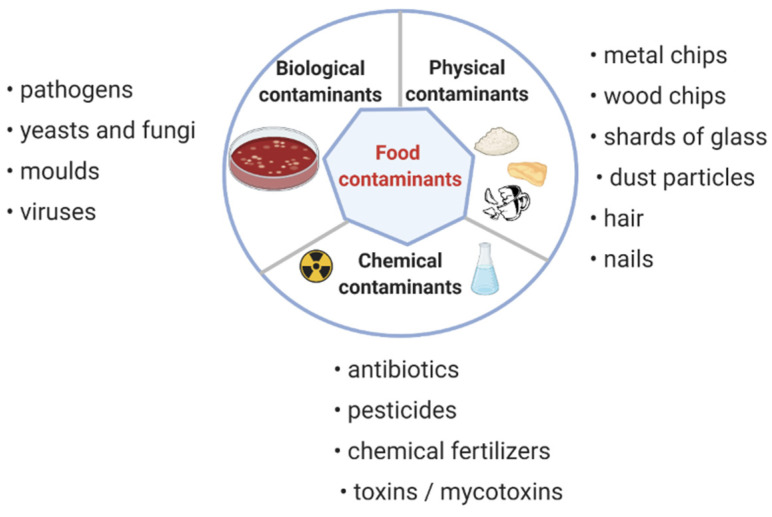
Contaminations in the food.

Everything we eat that is not harmful to animals and humans is labeled “safe food”. An organization in every country is responsible for food safety, regulating additives and their concentrations permitted in food [41]. Toxic compounds may be naturally occurring or by-products resulting from processing, storage, or cooking [42]. It is difficult to test for intoxications because most foods cannot be tested for every possible toxic compound. To accurately detect unknown and known contaminants, it is necessary to run several follow-up cases of intoxication [40]. Currently, the authorities need to be more concerned about food safety because globalization, easy traveling, and rapid food habits are changing. The illnesses caused by pathogens, toxins, and other contaminations in the food (Figure 2) cause a real health risk to humans and animals. Analyses and control measures mean a significant budget loss for the food industry [41]. 

A constant preoccupation of the food safety authorities is the exposure levels of the population or specific group of people, resulting in regulations that assess the maximum level of exposure allowed. Several studies discuss the toxicokinetic and toxicodynamics interaction of toxic compounds with the human body [43]. These studies reveal various detoxification strategies for reducing, annihilating, or converting toxic compounds. These strategies can be classified into physical (peeling, heat, ultraviolet light, ionizing radiation, and solution absorption), chemical (chlorination, oxidant, and hydrolytic substances utilization), and biological (inside the body or in food products using enzymes or probiotics). Because physical and chemical methods have some disadvantages associated with nutritional degradation, inefficiency for some toxins, secondary contaminants, and consumers’ acceptance and concerns, as well as a need to reduce and replace chemical technology with high sensitivity, specificity, and environment-friendly methods, biodetoxification using probiotics is proposed [6,44]. 

### 4.1. Chemical Contaminants and Their Impact on Human Health

#### 4.1.1. Heavy Metals

Human daily activities release heavy metals into the soil, air, and water. The most studied and known damage produced by heavy metals is the induced oxidative stress, resulting in cellular damage. Each heavy metal has its free radical generation mechanism targeting proteins involved in the apoptosis, cell cycle regulation, growth and differentiation, DNA methylation, and DNA repair—materializing in carcinogenesis. Some heavy metals may have neurotoxic impact induced by mechanisms, such as reducing neurotransmitters or accumulating mitochondria of neurons that disrupts adenosine triphosphate (ATP) synthesis [6,45]. 

Overwhelming metal contamination is a critical issue within the food industry, which undermines human health. The most common heavy metal contaminants are arsenic (As), cadmium (Cd), copper (Cu), mercury (Hg), nickel (Ni), lead (Pb), and zinc (Zn) [41]. Heavy metals entering the body increase the risk of developing cardiovascular, kidney, and neurological diseases [6,45]. A more toxic form of Hg is methylmercury, a strong neurotoxin, which affects the human central nervous system [46]. Pb is mutagenic and teratogenic, and it can negatively affect the neurotic system, interfering with the synthesis of hemoglobin, damaging kidney functionality, and reducing semen quality [47]. Cd can cause various diseases, such as cardiovascular, liver, reproductive system disorders, osteomalacia, and lung and renal cancer [48]. 

Among the detection methods for heavy metals, the most utilized are atomic absorption spectrometry, atomic fluorescence spectrometry, or spectrophotometry; lately, due to a demand for real-time detection, electrochemical biosensors have been used in this sense [6]. Bacterial biomass can also remove heavy metals from aqueous solutions [46]. 

#### 4.1.2. Acrylamide

Foods subjected to heat treatments (roasting and baking) undergo several unwanted changes, such as lipid oxidation, protein denaturation, vitamin degradation, and the formation of compounds harmful to the human body [49].

Acrylamide is mainly found in carbohydrate-rich bakery products (bread, biscuits, cookies, and baby foods based on cereals), french fries, chips, coffee, and meat preparations, subjected to high heat treatments. Small quantities of acrylamide, almost undetectable, can also be found in packaged foodstuffs due to its migration from the materials used in packaging that directly contact the product [49,50]. It is formed due to reactions between asparagine and reducing sugars (glucose, glyoxal, glycerol, and 2-deoxyglucose) [51]. The most conclusive detection method is mass spectrometry combined with capillary electrophoresis, gas, or liquid chromatography, especially high-performance liquid chromatography [50].

After ingestion, the human and animal bodies absorb and accumulate it in various organs, such as the heart, brain, liver, thymus, kidneys, muscle tissue, skin, and testes. The main pathway of acrylamide metabolism involves conversion to glycidamide and its conjugation to glutathione [49,50].

Studies have shown that acrylamide can cause genetic and reproductive toxicity, neurotoxicity, carcinogenicity, oxidative stress, and changes in genetic material (Khorshidian et al., 2020). When ingested doses are higher than recommended (100 mg/kg), it can cause acute toxic effects and lethal effects when it exceeds 150 mg/kg [49]. Probiotic capacity to reduce the damage produced by acrylamide ingestion is associated with their antioxidant activity [52].

#### 4.1.3. Polycyclic Aromatic Hydrocarbons—Benzo[a]Pyrene 

The primary source of polycyclic aromatic hydrocarbons (PAHs) is the incomplete combustion of the material’s body, such as coal, oil, and wood. PAHs are toxic to aquatic life, birds, and soil. They are absorbed by mammals through various methods (inhalation) and by plants through roots, which afterward translocate them to other parts of the plant. The most toxic member of the environmental pollutant PAHs family is Benzo[a]pyrene (B[a]P). B[a]P absorptions have pro-inflammatory effects and can induce tumors (gastrointestinal, bladder, and lung cancers), reproduction disorders, mutagenesis, disturbing development, and immunity deficiency [53,54,55].

Studies established that contamination with B[a]P is inevitable, which is caused by polluted water, soil exposure, and food consumption. Due to its low water solubility, B[a]P is recalcitrant to microbial degradation [54].

### 4.2. Biological Contaminants and Their Impact on Human Health 

#### Fungi—Molds and Yeasts and Their Mycotoxins

Probiotics are studied to improve food security and human health by inhibitory action on fungi—yeasts and molds. Around 5–10% of the world’s food system is affected because of fungal impairment causing carcinogenesis through the produced mycotoxins. As a result, many acids, including acetic, propionic, sorbic, lactic, and benzoic are used in food preservation. Concerns are raised because yeasts and molds developed a resistance to antibiotics, preservatives, and sanitizer agents, demanding a better alternative [56,57,58]. Among contaminants, mycotoxins are probably the biggest threat to human health due to their high carcinogenesis. In addition, mycotoxins formed by certain kinds of fungi can cause acute poisoning and a significant deficit in the immune system [59,60].

The interaction between mycotoxins and probiotic cells is influenced by the cellular wall’s integrity, which is responsible for the absorption capacity [61].

## 5. Biodetoxification Activity of Probiotics

Producers, authorities, and consumers face food safety-related challenges. The population is exposed to fungus, mycotoxin and virus infections, chemicals (acrylamide, benzopyrene, heavy metals), mutagenic and carcinogenic compounds. The need for viable, generally accepted, and applicable detoxification methods are sustained not necessarily by economic damage but by the danger to human well-being in general [37,41]. 

Biodetoxification may be an intrinsic phenomenon, mainly in the enzymatic system and human microbiota, but it can also be an external, controlled, and directed phenomenon that ensures food safety before the contaminated food product is ingested [4].

Probiotics can bind mutagens and carcinogens, such as aflatoxins [4]. 

Further, we will discuss how the most commonly used probiotic genera reported as being able to biodegrade, absorb, or induce physical adhesion to different toxic compounds or pathogen microorganisms are frequently incriminated for foodborne diseases. 

### 5.1. Lactobacillus (LAB) Genera and Their Biodetoxification Capacity

*Lactobacillus* genera are the most known and used probiotic [16,53]. For the conversion of glucose to lactic acid, Gram-positive and non-spore-forming bacteria, such as Lactic Acid Bacteria (LAB), can be used to initiate lactic acid fermentation [16]. During fermentation, lactose to lactate conversion reduces the danger of carcinogenic and mutagenic chemicals [62]. LAB can also remove pollutants from food through various metabolic activities, according to recent research (Table 1). Fermentation, antibiosis, and the capacity of the microbial cell wall to attach to the toxin are factors in these microorganisms’ decontaminant action [57]. The antimicrobial qualities of LAB can effectively limit the growth of other pathogenic microorganisms and fungi [54]. Yeast and lactic acid bacteria (LAB) mycotoxin involve fighting binding aflatoxins [63]. 

LAB reduces AFM1 (aflatoxin M1) and potentially decreases toxins in yogurt to a safe concentration for consumption (below 0.05 µg/kg). Research proved the capacity of *L. acidophilus* to bind AB1 and AM1 in cow’s milk [64]. A simulated gastrointestinal model sustained these results by proving the ability of *L. acidophilus* and *L. casei* (~10 log CFU/mL) to bind with AFB1 (aflatoxin B1); however, in contrast, it also underlined a reduced binding capacity in the presence of milk. The authors of the study concluded that micronutrients present in milk have a protective effect on the micotoxin (covering effect) [65]. Another study revealed that *L. kefiri* FR7 can reduce *Aspergillus flavus* and *A. carbonarius* growth and their mycotoxin production capacity [57].

Wu and his colleagues examined the prevention of colorectal carcinoma induced by B[a]P. They administered to mice, with colorectal-induced tumorigenesis, polymethoxiflavone (PMF), an anticancer agent found in citrus peels. The result indicated that by the oral administration of PMF, B[a]P-induced colon tumorigenesis (Benzo[a]pyrene) was blocked [66]. A practical explanation is gut microbiota modulation by prebiotic-like compounds. 

In 2021, a group of researchers proved that *L. acidophilus* NCFM (1  ×  10^10^ CFU/mL), among five bacterial strains (*L. plantarum* 121, *Leuconostoc mesenteroides* DM1-2, *L. acidophilus* NCFM, *L. paralimentarius* 412, and *L. pentosus* ML32), has the best capacity to bind B[a]P, the pH being (optimal pH 6) the parameter that influenced its binding yield the most, among incubation time, temperature, and strain concentration, [53]. Madreseh et al. proved that *L. fermentum* 1744 (ATCC 14931) (1 × 10^9^ CFU/mL) may significantly reduce heavy metal (Pb, Zn, Ni, and Cd) absorption and accumulation in living organisms (rainbow trout). The best results were obtained for the encapsulated probiotics in the presence of lactulose (10 g/kg feed) [45]. The probiotic’s ability to reduce heavy metals as well as the toxic effects of heavy metals in vitro and in vivo is related to its mechanism’s binding ability due to the numerous negatively charged functional groups found in the probiotics cell wall [67], the modulation of different over-expressed genes upon exposure to heavy metals [6], and an enhancement in the fecal excretion of ingested heavy metals [68].

To sum up, the two hypotheses are attributed to the probiotic detoxification action. The first mechanism consists of the physical connection between the probiotic and contaminant. The second is when probiotics and strains can mitigate the carcinogenic danger through their metabolism. The cell wall of probiotics is primarily composed of peptidoglycan found in glycan chains consisting of alternating N-needles tilglucosamine and N-2 acetylmuramic acid, linked by β-1,4 bond [74]. 

Factors affecting contaminants’ LAB bindings are associated with the proper selection of strains with a high capacity to eliminate the food contaminant [53,54,69]. We can state that the growth phase, incubation time, pH, contaminant concentration, and characteristics significantly affect probiotics’ binding/antimicrobial properties, but the binding ability may be related to the dose [54,75,76,77]. All studies showed that the detoxification rate is influenced by the contaminant and probiotic cell concentration, exposure time, pH, temperature, and nutrient presence [4,65,78,79]. 

### 5.2. Bifidobacteria Genera and Their Biodetoxification Capacity

The genus *Bifidobacterium* includes Gram-positive, non-motile, non-spore, Y- or V-shaped, anaerobic bacteria that produce lactic and acetic acids without producing CO_2_. *Bifidobacterium* growing temperature is around 36 °C and 38 °C, with optimum pH values ranging from 6.5 to 7. Amino acids, thiamin, and riboflavin can all be synthesized by *Bifidobacterium* [80,81]. Antibiotic resistance is a feature of select LAB, which has been widely employed to manufacture probiotic-fermented foods, called preparations. Short-chain fatty acids (SCFAs) interact with the host cell and gut microbiota as significant products of substrate fermentation in the gut [16]. The pH of the gut is lowered by these two acids, notably in the cecum and ascending colon. Many dangerous bacteria are suppressed in a low-pH environment; hence *Bifidobacterium*’s capacity to raise the acidity of the gut likely plays a role in its probiotic benefits [81]. Studies have indicated the binding or physical absorption of toxins by *Bifidobacterium* [42,55,70] (Table 2). A research paper discovered that having *B. lactis* HN019 in the diet can boost natural immunity. In macrophage cell lines, live or heat-killed *Bifidobacterium* and *Lactobacillus* species and certain of their cellular constituents can increase the generation of nitric oxide, hydrogen peroxide, cytokines tumor necrosis factor-, and interleukin-6 [80]. The binding ability of protoplasts and cell-free extract of three strains was determined in another study, revealing that the cell membrane was not the primary binding site and that B[a]P is not eliminated by metabolism. These observations highlight the relevance of cell wall preservation in B[a]P binding and support the existence of a cell wall-related physical phenomena that opposes metabolic breakdown [55].

*Bifidobacterium*’s potential mechanisms of detoxifications are therefore linked to their ability to bind the toxic compounds due to the presence of peptidoglycan and polysaccharides in the cell wall [82]. As in the *Lactobacillus* cases, the incubation time and viability -cell wall integrity strongly affects their biodetoxification ability [55,82]. 

### 5.3. Probiotic Yeasts and Their Biodetoxification Capacity

The genus *Saccharomyces*, more exactly *S. cerevisiae* strain is the most widely used for baking, alcoholic fermentation, and nutritional supplements for people and animals. Due to their inclusion in the Generally Recognized as Safe group, these species, together with LAB, offer an appropriate starting point for finding strategies to decrease food and human exposure to chemical contaminants [69,73,86,87]. Some yeast species (Table 3) have been used as biocontrol agents to prevent mycotoxin-producing filamentous fungus from growing on crops, food, and feed [86]. These species might help preserve agricultural goods and decrease mycotoxin contamination. In different technological processes, yeasts can be used for their direct inhibitory impact on pollutants, particularly mold toxin generation, independent of their growth-inhibiting effect [69]. Several yeast species’ cell walls can also bind mycotoxins from agricultural goods, successfully sanitizing them. Mycotoxicosis in cattle is also treated using probiotic yeasts or foods containing yeast cell walls or other ingredients. Yeasts are also known to have additional beneficial properties, such as breaking poisons into less harmful or even non-toxic forms [72]. Yeasts and their biotechnologically necessary enzymes may be sensitive to particular mycotoxins, posing a severe challenge to the biotechnological field, but unfortunately, yeast–mycotoxin interactions have been seriously understudied [70,86,88]. Filamentous fungus development and/or decreased gene expression involved in mycotoxin production can be limited by the yeast-generated metabolites. The main volatile organic compounds generated by *Pichia anomala* (fungi biocontrol agent), 2-phenylethanol (2-PE), have been shown to hinder spore germination and toxin production; in other words, biosynthesis was suppressed [87]. Yeast’s capacity to bind ochratoxin A increased during fermentation with two *Saccharomyces* strains by the addition of anthocyanin [86].

The yeast cell integrity seems to be the most important factor that influences the efficacity of yeast-related biodetoxification. Namely, cell surface areas, volume, cell wall thickness, and the presence of O–H/N–H bonds of proteins, polysaccharides, and 1,3-β-glucan from the yeast cell walls [87,89,90]. Parameters such as yeast exposure time, yeast concentration, initial toxin concentration, and temperature are the ones mentioned by the scientific literature as being influencing factors in the biodetoxification process [87,91]. In contrast to *Lactobacillus*, yeast’s capability to bind mycotoxins is not significantly influenced by cell viability. The main condition for the inactive yeast cells was the cell wall wholeness. Destroyed yeast cells proved with almost 50% less biodetoxification capacity [90].

### 5.4. Other Probiotics or Promising Probiotic Candidates and Their Biodetoxification Capacity

Several probiotic species and promising probiotic candidates (Table 4) have different biodetoxification activities. Probiotics may use several mechanisms (such as epoxidation, hydroxylation, dehydrogenation, and reduction) or metabolites (antimicrobial proteins) for the toxins’ degradation [88,93]. Bacitracin A, for example, is a non-ribosomal peptide antibiotic developed by *Bacillus licheniformis* strain HN-5 with high antibacterial activity. *Bacillus* spp. are rod-shaped, Gram-positive, endospore-forming organisms that can be obligate aerobes or facultative anaerobes and are a potent antibiotic against Gram-positive and -negative bacteria. The *bacABC* operon and *bacT*, which encode non-ribosomal peptide synthetase and thioesterase, respectively, make up the bacitracin synthetase gene cluster in *B. licheniformis.* Commercially *B. licheniformis* is utilized in the manufacture of bacitracin, an extensively used animal feed. The processes behind bacitracin’s ability to reduce infectious illnesses in animals have previously been studied [94].

Often utilized in producing industrial enzymes, including amylase and protease, *Bacillus licheniformis* is a common bacteria found in soil and waste organic material. According to a prior study, several strains of *B. licheniformis* have a lot of promise as probiotics or nutrition supplements for humans [93]. After 36 h of incubation, *B. licheniformis* CK1 reduced ZEN by 95.8% in *Lactobacillus* broth by degradation of the mycotoxin (the HPLC chromatogram *B. licheniformis* CK1 cell wall revealed no ZEN). The authors believe that the extracellular xylanase, cellulase, and protease produced by *B. licheniformis* CK1 are responsible for the degradation. According to the data, ZEN at a concentration of 2 ppm was not harmful to *B. licheniformis* [95].

Not so commonly used probiotic strains, such as *Pediococcus acidilactici* RC005 and *P. pentosaceus* RC006, absorbed between 26% and 34% of aflatoxin M1 from milk, from a concentration of approx. 30 and 34 ng/mL. The authors also discussed the desorption phenomena observed in 100% of the tested yeasts strain [88]. 

Future studies need to sustain the less-studied probiotic genera’s (other than *Lactobacillus*, *Bifidobacterium,* and *Saccharomyces*) biodetoxification capacities and elucidate their mechanisms of action.

## 6. Probiotic Antimutagenic Activity

It has been proven that genotoxic substances and antibiotics created in the human body can induce genetic mutations and carcinogenesis [99]. As a solution to this effect, it is recommended to use antimutagens to prevent genetic mutations transmitted by some foods, cancers, or tumors. Antimutagenics are substances that can reduce the occurrence of mutations at the cellular level, acting on DNA replication and repair [100]. Antimutagens use chemical or enzymatic pathways to annihilate mutagens’ actions. 

The autochthonous microflora in the human GI tract is wide-open to genotoxic compounds at high frequency. Some bacteria in the gut can efficiently bind mutagenic pyrolysates to decrease their mutagenicity. *Bifidobacteria* are among the more significant bacteria in the human gut with this effect [34]. They are used as probiotic dietary supplements.

It was also demonstrated that probiotics could act as immunomodulators by influencing the gut-associated lymphoid tissue distributed throughout the GI tract [101]. Additionally, literature reports that probiotics can produce butyric and acetic acids with antimutagenic activity (can fight chemical mutagens or promutagens). Thus, these properties are associated with the consumption of viable and able-to-colonize probiotic cells. Compounds that diminish the effects of the mutagen are classified as desmutagens or bioantimutagens. Desmutagens act in a chemical or enzymatic direction by inducing inhibition of the mutagens’ activity. Meanwhile, bioantimutagens act on DNA replication and inhibit the effects of the mutagen [102]. 

Another side of probiotics and their antimutagenic effect is how they could be introduced into humans’ diets in an effective manner. Thus, a key characteristic of probiotics present in functional foods is viability. Among these types of functional products is yogurt. Yogurt is an excellent matrix used for probiotics delivery, with the mention that it should contain a minimum number of 10^6^ CFU/g probiotics at the time of use. Several factors such as pH, water activity, oxygen, strain type, and other strains influence this [11,16]. The adverse effects of probiotics could be minimized by different strategies, such as microencapsulation of probiotics, the addition of enzymes, and prebiotics [103]. 

DNA alteration and carcinogenesis may be induced by the increase in mutagens and promutagens in the system [102]. Scientists have proven that butyric and acetic acids, of probiotic nature, have a broad antimutagenic activity. Thus, GI disorders may be reduced using probiotics, which can avoid the hazard of DNA genotoxins. Probiotics act as immunomodulators by influencing the gut-associated lymphoid tissue distributed throughout the GI tract. To have a positive impact on human health, probiotic cells need to be able to colonize the intestine. For *L. acidophilus* and *Bifidobacterium* spp., their products of fermentation are probiotic bacteria that provide antimutagenic and anti-carcinogenic activities. 

It has been reported that activating carcinogenic enzymes, such as nitroreductase, β-glucuronidase, and azoreductase, are inactivated or *L. acidophilus* reduces their activity [104]. 

By fermenting milk with different *Lactobacillus strains* to obtain the yogurt, more peptides are formed, which present various bioactive compounds. These compounds have positive effects on consumer health, namely antimutagenic and antioxidative effects. Simultaneously, bioactive compounds are used to create functional foods and increase some foods’ shelf life through the antioxidant effect [105]. 

*L. paracasei* subsp. tolerance JG22 also has a positive effect on the control of compounds that can express mutagenesis. There are some proven valuable characteristics of this strain, namely, high resistance to an acid environment (pH 2.0) and bile salts (0.5%), resistance to different antibiotics, and an adequate ability to colonize the gut [102]. The authors conclude that *L. paracasei* subsp. tolerance of JG22 is an excellent probiotic to be included in functional foods to prevent colon mutagenesis or tumorigenesis [102,106]. Therefore, only viable probiotic cells can inhibit or bind mutagens. 

## 7. Anti-Carcinogenic Effect of Probiotics

Cancer is a pathology caused by multiple triggers. Our World in Data reports cancer as the second cause of death worldwide [107]. Food carcinogens formed in foods cooked at high temperatures and inadequately stored or contaminated with raw materials (heterocyclic amines (HCA), polycyclic aromatic hydrocarbons (PAH), mycotoxins (aflatoxins), N-nitroso compounds, acrylamide, and heavy metals) increase the potential risk factors for cancer [61,100]. In the GI tract, probiotics connect and degrade carcinogenic compounds [108,109]. The cell wall of probiotics may be an essential factor in binding free toxins in the intestine [104].

Factors, such as genetic predisposition, personal diet, lifestyle, physical activity, obesity, type 2 diabetes, abusive alcohol use, inflammation, and smoking, significantly influence carcinogenesis [110]. Several studies have confirmed that some opportunistic microorganisms, such as *Bacteroides fragilis*, *Fusobacterium nucleatum*, *Helicobacter hepaticus*, *Streptococcus bovis*, and *E. coli*, may indicate different types of cancer [7].

There are several pathways attributed to the anti-carcinogenic effect of different probiotics (Table 5). Among these, the most stated ones are the alteration and deactivation of carcinogens or mutagens, decreasing pH of the gut environment regulating the gut microflora and suppressing the growth of carcinogenesis microbiota, immunomodulatory properties (such as increased peripheral immunoglobin production, stimulation of IgA secretion, and decreased pro-inflammatory cytokine production), modulation of apoptosis (through SFCA production, and glutathione transferase activity stimulation), sustain cancer cell differentiation (through butyric acid action), inhibition of the tyrosine kinase signaling pathway, and DNA protection from oxidation [19,80,111,112]. Cancer cell proliferation is inhibited by probiotic action by making the cells more susceptible to apoptosis [8]. These mechanisms involve activation of pro-caspases, decreasing the anti-apoptotic Bcl-2, and increasing the sensitivity of pro-apoptotic Bax proteins. 

The scientific literature reveals that living or dead probiotic cells, their components (cell wall, peptidoglycan, and cytoplasmic fraction), or metabolites (exopolysaccharides, SCFA) can produce substantial antiproliferative effects in cancer cell lines [81,113].

**Figure 3:**
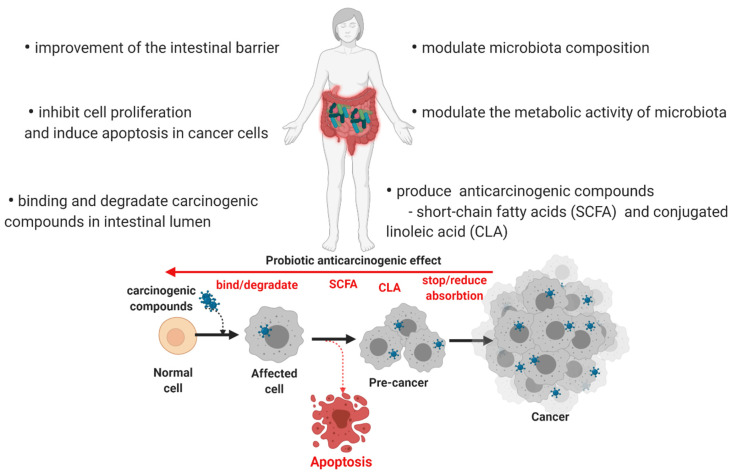
Anti-carcinogenic mechanisms of probiotics.

Figure 3 describes the probiotic mechanisms reported in the scientific literature responsible for their anti-carcinogenic activity.

## 8. Conclusions and Perspectives

This paper focuses on the protective and biodetoxification capacities of different probiotic strains. Probiotics are popular for their role in different pathologies, mostly in the intestinal related ones. Their impact on gut microorganisms is crucial because they can positively (i.e., biodetoxification from mycotoxin, fungi, acrylamide, metals, virus, reduce pro-inflammatory responses, antimutagenic and anti-carcinogenic activities) and negatively (i.e., transfer of antibiotic resistance) modulate human health. Considering that consumers respond differently to probiotics according to age, genetic characteristics of gut bacteria, diet, antibiotic use, and environmental cues, precautions are necessary before their use, and for this reason, they should be recommended only by health care personnel/clinicians, while more concerns are in their market distribution.

The biodetoxification mechanisms of the action of probiotics belonging to *Lactobacillus*, *Bifidobacterium*, *Saccharomyces*, and other types of more or less popular genera (*Bacillus*, *Enterococcus*, *Escherichia*, *Streptomyce*, *Pediococcus*) are proved to be influenced by factors specific to (i) bacteria genus and strain; (ii) environmental dependent factors; and (iii) toxin dependent factors. Probiotics belonging to *Lactobacillus* are the most studied ones and are more correlated with the ability to bind toxins (mycotoxins, heavy metals, etc.) on the cell wall. Reactive functional groups and compounds present in the cell wall, such as proteins, peptidoglycan, and polysaccharides; 1,3-β-glucan for the yeast cell wall, are recognized to be responsible for probiotic binding capacity. The differences between the strains in relation to toxin absorption and binding are given probably due to the diversity in cell wall structures and bacterial cell membranes. 

Other probiotic biodetoxification pathways are correlated with probiotic metabolites, co-cultivation of different probiotics or different probiotic/compound formulations (i.e., lactulose), gene expression, and sustaining fecal excretion. 

Due to the fact that probiotics decrease toxin absorption and by reducing its toxicity, they are correlated with strong anti-carcinogenesis and anti-mutagenesis action. 

Based on a thorough review of the capacity of probiotics to react with pathogens, fungi infection, mycotoxins, acrylamide toxicity, benzopyrene, and heavy metals, we conclude that specific probiotic strains and combinations offer significant health outcomes and positively impact in vitro and in vivo detoxification processes. Despite the fact that there are many publications on the biodetoxification properties of probiotics, their application in practice in the detoxification of food and/or feed has been narrow. To increase this utilization, we concluded that specific mechanism pathways should be elucidated, the toxicity of degradation products should be also studied, and there should be safety regulation on the use of probiotic strains towards food matrices and in vivo systems.

## Figures and Tables

**Table 1:** *Lactobacillus* strains, their biodetoxification mechanisms, and rates for different food contaminants.

*Lactobacillus* Strain/mix	Cell Count CFU/mL	Contaminant	Food/Environment	Contaminant Level	Biodetox. Mechanism	Detox. Rate	Ref.
*L. acidophilus* ATCC 4356 *L. casei* ATCC 39392	10^9^	AFB1	milk in vitro digestion	5 µg /mL	absorption enzymatic degradation	14–70%	[65]
*L. acidophilus* ATCC 9224, 4356, CECT 4529, CECT 4179 *L. acidophilus* CNRZ 55, 217*L. brevis* ATCC 14869 *L. brevis* DSMZ 1268 *L. casei* CECT 5275 *L. crispatus* M247, DSMZ 20584 *L. plantarum* WCFS1 *L. rhamnosus* ATCC 53103	OD10	As	in vivo—NCM460 colon epithelium cell HT29-MTX mucosecretory adenocarcinoma cells cooked white rice	30 mg/kg	binding	1–6%	[69]
*L. acidophilus* La-5	10^8^	AFB1 AFM1	cow milk	1 µg /mL 100 µg /mL	↓ bioaccessibility	13.53–35.53% 17.65–71.52%	[64]
*L. acidophilus* EMCC 1324	1, 5, 7 × 10^9^	AFB1, AFB2, AFG1, AFG2	in vitro in vivo (rats)	50 µg /mL	binding absorption	95.59%	[70]
*L. acidophilus* (isolated from traditional dough)	10^6^	*S. aureus* ATCC 25923, Si. dysenteriae	Mueller–Hinton agar	10^6^ CFU/mL	growth inhibition	12.1 mm 20.9 mm 14.7 mm	[2]
*L. acidophilus* NCFM	10^8 9 10^	B[a]P	in vitro	1.0 μg/mL	absorption	45–60%	[53]
*L. brevis* LN871494 *L. kefiri* MH107106	10^8^	*A. flavus* *A. carbonarius*	in vitro (agar)	10^6^ spores/mL	growth inhibition—antifungal activity	20–50%	[57]
*L. bulgaricus* KLDS1.0207	10^10^	Pb	in vivo (BALB/c mice)	50 mg/kg/day	binding excretion	↑ Pb excretion	[71]
*L. reuteri* CGMCC 1.3264	10^8^	ZEN	maize kernels	5 mg/L	hydrolysis	100%	[59]
*L. fermentum* 1744 ATCC 14931	10^9^	Heavy metals Pb, Zn, Ni, Cd	living fish (rainbow trout)	-	stop accumulation (vs. control)	na	[45]
*L. delbrueckii* subsp. bulgaricus DSM 20,081 *L. sakei* subsp. sakei DSM 20,017 *L. rhamnosus* DSM 20,021 *L. plantarum* subsp. plantarum PTCC 1896	10^7^	Acrylamide	whole-wheat bread	47.6 µg /kg	↓ Maillard reaction and acrylamide formation due to fermentation	85.5%	[72]
*L. paracasei* LOCK 1091 *L. pentosus* LOCK 1094 *L. plantarum* LOCK 0860 *L. reuteri* LOCK 1092 *L. rhamnosus* LOCK 1091	4.5 × 10^10^	AFB_1_	in vivo—broiler chickens	1 mg/kg 5 mg/kg	↑ excretion	41–68%	[73]
*L. plantarum* Bacillus coagulans	10^9^	Hg	in vivo—Wistar rats	20 μg/mL of mercuric chloride	↑ elimination	>50%	[46]
*L. plantarum* PTCC 1058, LP3, AF1, LU5	1.6 × 10^5^	*A. flavus* PTCC 5004 *A. parasiticus* PTCC 5018 *A. nidulans* PTCC 5014 *A. ochraceus* PTCC 5060	ice cream		growth inhibition	27.6 ± 0.9 mm	[3]
*L. plantarum* PTCC 1058, LP3, AF1, LU5	1.6 × 10^5^	AFM1 OTA	ice cream	0.5 µg /kg 0.5 µg /kg	degradation	26–52% 32–58%	[3]

↓—decrease, ↑—increase, ZEN—zearolenone mycotoxin; AFB, AFM1—aflatoxin B1, M1; OTA—ochratoxin A; Pb—lead; Zn—zinc; Ni—nickel; Cd—cadmium; Hg—mercury, As—arsenic; B[a]P—benzo[a]pyrene, A—*Aspergillus*, L—Lactobacillus, S—*Staphylococcus*, Si—S*higella.*

**Table 2:** *Bifidobacteria* strains, their biodetoxification mechanisms, and rates for different food contaminates.

*Bifidobacteria* Strain/Mix	Cell Count CFU/mL	Contaminant	Food/Environment	Contaminant Level	Biodetox. Mechanism	Detox. Rate	Ref.
*B. animalis subsp. Lactis BI-04*	5 × 10^8^	B[a]P	in vitro—colon epithelial cells in vitro digestion	0.5 µg /mL	markedly relieved cell damage	95%	[83]
*B. animalis subsp. Lactis* *B. longum subsp. Infantis* ATCC 15697 *B. longum subsp. Longum* ATCC 15707	10^8^	*Fusobacterium nucleatum* ATCC 25585 *Porphyromonas gingivalis* 33277 *Streptococcus oralis*	in vitro	10^6^ CFU/mL	DNA-DNA hybridization	64.9%; 54%	[84]
*B. animalis* subsp*. Lactis* BI-04, 1.2226, and HN019 *B. bifidum Bb-02* *B. breve* 1.2213 and BD-01 *B. longum* subsp*. Infantis Bi26 and BY12,* *B. longum* subsp*. Longum* 1.2186	5 × 10^8^	B[a]P	in vitro	100 μg/mL	physical absorption xenobiotics biotransformation	78%	[55]
*B. bifidum*	1; 5; 7 × 10^9^	AFB1, AFB2, AFG1, AFG2	in vitro in vivo (rats)	50 µg /mL	binding absorption	95.59%	[70]
*B. bifidum DDBA*	1 × 10^11^	ZEN	in vitro	2.5 μg/mL	physical absorption biotransformation	98%	[42]
*B. bifidum NRRL B-41410*	2 × 10^6^	AFM1	in vivo	50 µg /mL	physical absorption metabolism mitigation	45.17%	[85]

AFB1—aflatoxin B1; AFB2, AFG1, AFG2, AFM1—aflatoxin B2, G1, G2, M1; ZEN—zearalenone; B[a]P—benzopyrene.

**Table 3:** Yeast strains, their biodetoxification mechanisms, and rates for different food contaminants.

Probiotic Yeasts Strain/mix	Cell Count CFU/mL	Contaminant	Food/Environment	Contaminant Level	Biodetox. Mechanism	Detox. Rate	Ref.
*Aureobasidum pullulans* L1	10^8^	acrylamide	fried potato	1600 μg/kg in the control	stop formation	83%	[92]
*Saccharomyces cerevisiae* CCTCC 93161	1.5 × 10^6^	PAT	fermentation broth	500 μg/L	physical adsorption	53.97% (6 h fermentation) 85.88% (24 h fermentation)	[87]
*S. cerevisiae* S10c *S. cerevisiae* S6u	10^6^	OTA	wine	2 µg/kg	absorption	29% white win 45.4–49.5% red win with extra anthocyanins	[86]
*S. cerevisiae* LOCK 0119	4 × 10^6^	AFB_1_	in vivo—broiler chickens	1 mg/kg 5 mg/kg	↑ excretion	41–68%	[73]
Kyokai 6 *S. cerevisiae* BY4743, VRB, Ultralevura, YPS128, UWOPS03–461.4	OD4	As	in vivo—NCM460 colon epithelium cell HT29-MTX mucosecretory adenocarcinoma cells cooked rice	30 mg/kg	binding	1–6%	[69]
*S. cerevisiae* ATCC 64712 *Kluyveromyces lactis* CBS 2359	1; 5; 7 × 10^9^	AFB1, AFB2, AFG1, AFG2	in vitro in vivo—rats	50 µg /mL	Binding absorption	95.59%	[70]
*S. cerevisiae RC016* *S. boulardii RC009* *Kluveromyces marxianus VM003*	10^7^	AFM1	in vitro	10 ng/mL	adsorption desorption	19, 25, 36% 100, 46, 100%	[88]

PAT—patulin; OTA—ocratoxin; AFB1, AFB2, AFG1, AFG2, AFM1—aflatoxin B1, B2, G1, G2, M1; As—arsenic; ↑—increase.

**Table 4:** Other probiotic strains or promising probiotic candidates, their biodetoxification mechanisms, and rates for different food contamination.

Other Probiotic/Probiotic Candidates Strain /Mix	Cell Count CFU/mL	Contaminant	Food/Environment	Contaminant Level	Biodetox. Mechanism	Detox. Rate	Ref.
*Bacillus licheniformis* strain JS (antimicrobial peptides)	50, 70, 100 μL	*B.cereus* *Shigella dysenteriae*	in vivo in vitro	nm	growth inhibition	21 mm B. cereus 14 mm S. dysenteriae	[93]
*B. licheniformis* HN-5	107–108	*Pantoea ananatis*	rice	10 / 40 µg/mL	growth inhibition	48.49 ± 0.15%/75.26 ± 0.15%	[94]
*B. licheniformis* CK1	Unknown	ZEN	in vitro	2.75 μg/mL	degradation	98%	[95]
*Enterococcus* strain *E. faecium* DUTYH_16120012	0.5 OD600	Pb	in vitro (MRS)	50 mg/L	removing	80.58 ± 1.65%	[96]
*Escherichia coli* Nissle 1917 (EcN)	2 × 10^3^	*Escherichia coli*	in vitro (Vero cells)	2 × 10^3^	↓ EHEC growth in co-culture ↓ stx2a expression	49.6% and 67.8% at 4 and 24 h of cultivation 2 and 5.4 fold at 4 and 24 h of cultivation	[97]
*E. coli* Nissle 1917 (EcN-2, EcN-22, EcN-23)	109	Cd Hg Pb	in vivo mice	1.6 ± 0.24 μg/mL	degradation	80% ↑ survival rate	[98]
*Streptomyces cacaoi* subsp. Asoensis K234	Unknown	AFB1	in vitro	1 μg/mL	degradation	88.34 ± 15.62	[4]
*Streptococcus thermophiles*	106	AFM1	in vitro	50 μg/mL	binding	58.5%	[4]
*Pediococcus acidilactici* KTU05-7, KTU05-8	9.2	Acrylamide	rye bread	-	↓ formation	38.33%	[51]
*P. acidilactici* RC005 *P. pentosaceus* RC006	107	AFM1	in vitro	10 ng/mL	adsorption desorption	34, 26% 33, 71%	[88]
*P. pentosaceus* TMU457	1010-15	AFB1	in vitro	5 µg /mL	binding	75.06 ± 1.60%	[4]
*P. pentosaceus* LN828199 *P. pentosaceus* LN871493	108	*A. flavus* *A. carbonarius*	in vitro (agar)	10^6^ spores/mL	growth inhibition—antifungal activity	20–50%	[57]

*B*—*Bacillus*; *P*.—*Pediococcu*; *E*.—*Escherichia*; ↓—decrease; AFB1, AFM1—aflatoxin B1, M1; ZEN—zearalenone; cadmium—Cd; mercury—Hg; lead—Pb; *A*.—*Aspergillus.*

**Table 5:** Most used probiotic strains and their impact on different cancers.

Probiotic Strain	Study Type	Cancer Type/Cell Lines/Carcinogen	Way of Action/Findings	Conditions	Ref.
*L. acidophilus* CICC 6074 S-layer protein	In vitro	HT-29 human CRC cells	↓ proliferation, chromatin condensation, nuclear fragmentation, induce apoptosis	25, 50, and 100 mg/L S-layer protein	[110]
*L. acidophilus CRL 636 + L. reuteri CRL 1101 + selenium*	In vitro	-	Preventive effect ↑ intracellular SeCys and SeMet	5 mg Se/L as selenite	[114]
*L. casei SR1, L. casei SR2, L. paracasei SR4* isolated from human breast milk	In vivo	HeLa cervix cancer cells	Sustain apoptosis by ↑ the expression of apoptotic genes *BAX*, *BAD*, *caspase3*, *caspase8*, and *caspase9* ↓ expression of *BCl-2* gene, ↓ proliferation	1.0 × 10^7^ to 1.0 × 10^8^ CFU/mL	[115]
*L. casei CRL431*	In vivo In vivo	4T1 breast cancer cells BALB/c mice	Improve the capecitabine’s toxicity on 4T1 cells ↓ capecitabine side effects ↓ intestinal mucositis and mortality ↓ decreased IL-6 ↑ immune response	1 × 10^9^ CFU/mL 36-day experimental protocol	[116]
*L. debrueckii* spp. *bulgaricus* LB-G040	In vivo	Colitis-associated cancer C57BL/6 mice	modulate inflammatory responses, inhibit tumor growth, ↓clinical signs of intestinal inflammation	3 times/week 1 × 10^9^ CFU by gavage	[117]
*L. fermentum* NCIMB 5221 *+ Lactobacillus acidophilus* ATCC 314	In vitro	CaCo2 adenoma cells	↓ proliferation induce apoptosis	*L. fermentum* 0.5 × 10^10^ CFU *L. acidophilus* 0.5 × 10^10^ CFU	[118]
*L. kefiri* LKF01 Kefibios ^®^	In vivo	76 patients with any solid tumor under therapy	↓ chemotherapy, radiotherapy, and immunotherapy side effects—diarrhea	5 drops/day (10^9^ CFU)	[119]
*L. helveticus* MB2-1 exopolysaccharides	In vitro	HT-29 CRC human cells	Induce apoptosis antiproliferative activity ↑ intracellular reactive oxygen species ↑ pro-apoptotic Bax and mitochondrial cytochrome c ↓ anti-apoptotic Bcl-2	0, 100, 200, 400 and 600 μg/mL exopolysaccharides	[120]
*L. plantarum I-UL4, TL1, RS5, RI11, RG11,* and *RG14* isolated from Malaysian food	In vitro	Cancer cells: MCF-7 breast CRC, HT-29 HeLa cervical Hep-G2 liver HL60, K562 leukemia	Antiproliferative and apoptotic effects on MCF-7 strain-specific and cell type-dependent cytotoxic effects no toxic effect or hemolysis on normal cells	*L. plantarum* added in conc. 0.47–30% (*v/v*)	[20]
*L. reuteri* FLRE5K1	In vivo	Melanoma cell line B16-F10 injected in 8-week-old female BALB/C mice	↓ melanoma occurrence ↑ survival rate	10^9^ CFU/mL/day, 7 days prior to and after melanoma injection	[121]
*L. rhamnosus* GC	In vivo	Gastric cancer-induced in male NMRI inbred albino mice	↓ tumor volume, size ↑ white blood cells no. ↑ level of Bax/Bcl-2 ratio improvement capecitabine chemotherapy	1 × 10^8^ CFU/100 µL saline/day	[122]
*B. bifidum* (isolated from infants’ feaces)	In vitro	SW742 human colon cancer cell line	Necrosis of the tumor cells	Probiotic growth in aerobic conditions, 1 × 10^5^ CFU application on cell	[80]
*B. longum* BB536-y *and fructooligosaccharides*	In vivo	Human colorectal cancer	Preventive action ↑ amount of SCFA ↓ *Bacteroides fragilis* enterotoxin production ↓ growth of putrefactive bacteria	1/day-5days BB536-y and BB536-y and FOS	[123]
*B. lactis* Bl-04 + *Lactobacillus acidophilus* NCFM	In vivo	CRC	Therapeutic action by microbiota modulation, ↑ butyrate-producing bacteria (*Faecalibacterium* and *Clostridiales* spp.)	2/day 1.4 × 10^10^ CFU *B. lactis*, 7 × 10^9^ CFU *L. acidophilus*	[109]
*Saccharomyces cerevisiae* PTCC 5052 –heat-killed	In vitro	CRC SW480 cell line	antiproliferative effect pro-apoptotic effect via Akt/NF-kB signaling pathway	1 × 10^6^ cell/mL heat-killed cells	[108]
*E. coli* Nissle 1917	In vivo	SMMC-7721 cancer cell injected into BALB/c nude mice	↓ tumor growth ↑ treatment response	5 × 10^6^ CFUs/100 μL	[98]
*Lactococcus lactis subsp. lactis isolate (R7)*	In vivo	Wistar rats Induced CRC	Anticancerigenic action ↓ intestinal morphological changes	1 mL bacterial suspension (10^8^ CFU/mL) 1/day, 6 weeks. by gavage	[124]

↓—decrease/downregulating; ↑—increase/upregulating, CRC—colorectal cancer, IL-6—cytokine related with bad prognosis in advanced cancers, SeCys—selenocysteine, SeMet-L—selenomethionine, CFU—colony formin units, SCFA—short-chain fatty acid, Bcl-2—B-cell lymphoma 2 with role in apoptosis regulation, Bax gene-modulates apoptosis.

## Data Availability

Not applicable.

## References

[1] Singhvi N., Gupta V., Gaur M., Sharma V., Puri A., Singh Y., Dubey G.P., Lal R. (2019). Interplay of Human Gut Microbiome in Health and Wellness. Indian J. Microbiol..

[2] Jabbari V., Mokarram R.R., Khiabani M.S., Askari F., Ahmadi E., Hassanzadeh A.m., Aghazadeh S.b., Asgharzadeh M., Kafil H.S. (2017). Molecular Identification of Lactobacillus Acidophilus as a Probiotic Potential from Traditional Doogh Samples and Evaluation of Their Antimicrobial Activity against Some Pathogenic Bacteria. Biomed. Res..

[3] Hashemi S.M.B., Gholamhosseinpour A. (2019). Fermentation of table cream by Lactobacillus plantarum strains: Effect on fungal growth, aflatoxin M1 and ochratoxin A. Int. J. Food Sci. Technol..

[4] Afshar P., Shokrzadeh M., Raeisi S.N., Ghorbani-HasanSaraei A., Nasiraii L.R. (2020). Aflatoxins biodetoxification strategies based on probiotic bacteria. Toxicon.

[5] Abdel-Shafy H.I., Mansour M.S.M. (2016). A review on polycyclic aromatic hydrocarbons: Source, environmental impact, effect on human health and remediation. Egypt. J. Pet..

[6] Abdel-Megeed R.M. (2020). Probiotics: A Promising Generation of Heavy Metal Detoxification. Biol. Trace Element Res..

[7] Vivarelli S., Salemi R., Candido S., Falzone L., Santagati M., Stefani S., Torino F., Banna G.L., Tonini G., Libra M. (2019). Gut Microbiota and Cancer: From Pathogenesis to Therapy. Cancers.

[8] Banna G.L., Torino F., Marletta F., Santagati M., Salemi R., Cannarozzo E., Falzone L., Ferraù F., Libra M. (2017). Lactobacillus rhamnosus GG: An Overview to Explore the Rationale of Its Use in Cancer. Front. Pharmacol..

[9] Sharifi-Rad J., Rodrigues C., Stojanović-Radić Z., Dimitrijević M., Aleksić A., Neffe-Skocińska K., Zielińska D., Kołożyn-Krajewska D., Salehi B., Prabu S.M. (2020). Probiotics: Versatile Bioactive Components in Promoting Human Health. Medicina.

[10] Lee E.-S., Song E.-J., Nam Y.-D., Lee S.-Y. (2018). Probiotics in human health and disease: From nutribiotics to pharmabiotics. J. Microbiol..

[11] Min M., Bunt C.R., Mason S.L., Hussain M.A. (2018). Non-dairy probiotic food products: An emerging group of functional foods. Crit. Rev. Food Sci. Nutr..

[12] Hill C., Guarner F., Reid G., Gibson G.R., Merenstein D.J., Pot B., Morelli L., Canani R.B., Flint H.J., Salminen S. (2014). Expert consensus document: The International Scientific Association for Probiotics and Prebiotics consensus statement on the scope and appropriate use of the term probiotic. Nat. Rev. Gastroenterol. Hepatol..

[13] Salminen S., Collado M.C., Endo A., Hill C., Lebeer S., Quigley E.M.M., Sanders M.E., Shamir R., Swann J.R., Szajewska H. (2021). The International Scientific Association of Probiotics and Prebiotics (ISAPP) consensus statement on the definition and scope of postbiotics. Nat. Rev. Gastroenterol. Hepatol..

[14] Vinderola G., Sanders M.E., Salminen S. (2022). The Concept of Postbiotics. Foods.

[15] Ohland C.L., Macnaughton W.K. (2010). Probiotic bacteria and intestinal epithelial barrier function. Am. J. Physiol. Liver Physiol..

[16] Pop O.L., Socaci S., Galanakis C.M. (2019). Chapter 10—Pro and Prebiotics foods that modulate human health. The Role of Alternative and Innovative Food Ingredients and Products in Consumer Wellness.

[17] Coman V., Vodnar D.C. (2020). Gut microbiota and old age: Modulating factors and interventions for healthy longevity. Exp. Gerontol..

[18] Johnson E.M., Jung Y.-G., Jin Y.-Y., Jayabalan R., Yang S.H., Suh J.W. (2017). Bacteriocins as food preservatives: Challenges and emerging horizons. Crit. Rev. Food Sci. Nutr..

[19] Azad A.K., Sarker M., Wan D. (2018). Immunomodulatory Effects of Probiotics on Cytokine Profiles. BioMed Res. Int..

[20] Chuah L.-O., Foo H.L., Loh T.C., Alitheen N.B.M., Yeap S.K., Mutalib N.E.A., Rahim R.A., Yusoff K. (2019). Postbiotic metabolites produced by Lactobacillus plantarum strains exert selective cytotoxicity effects on cancer cells. BMC Complement. Altern. Med..

[21] Kumar R., Sood U., Gupta V., Singh M., Scaria J., Lal R. (2019). Recent Advancements in the Development of Modern Probiotics for Restoring Human Gut Microbiome Dysbiosis. Indian J. Microbiol..

[22] Chang C.-J., Lin T.-L., Tsai Y.-L., Wu T.-R., Lai W.-F., Lu C.-C., Lai H.-C. (2019). Next generation probiotics in disease amelioration. J. Food Drug Anal..

[23] Ahtesh F.B., Stojanovska L., Apostolopoulos V. (2018). Anti-hypertensive peptides released from milk proteins by probiotics. Maturitas.

[24] Banerjee D., Jain T., Bose S., Bhosale V., Rani V., Yadav U.C.S. (2018). Importance of Probiotics in Human Health. Functional Food and Human Health.

[25] Amin N., Boccardi V., Taghizadeh M., Jafarnejad S. (2019). Probiotics and bone disorders: The role of RANKL/RANK/OPG pathway. Aging (Milan, Italy).

[26] Asha M.Z., Khalil S.F.H. (2020). Efficacy and Safety of Probiotics, Prebiotics and Synbiotics in the Treatment of Irritable Bowel Syndrome: A systematic review and meta-analysis. Sultan Qaboos Univ. Med J. [SQUMJ].

[27] Borges S., Silva J., Teixeira P. (2013). The role of lactobacilli and probiotics in maintaining vaginal health. Arch. Gynecol. Obstet..

[28] Bustamante M., Oomah B.D., Oliveira W.P., Burgos-Díaz C., Rubilar M., Shene C. (2019). Probiotics and prebiotics potential for the care of skin, female urogenital tract, and respiratory tract. Folia Microbiol..

[29] Hedin C., Whelan K., Lindsay J.O. (2007). Evidence for the use of probiotics and prebiotics in inflammatory bowel disease: A review of clinical trials. Proc. Nutr. Soc..

[30] Islam S.U. (2016). Clinical Uses of Probiotics. Medicine.

[31] Barouei J., Moussavi M., Hodgson D. (2015). Perinatal maternal probiotic intervention impacts immune responses and ileal mucin gene expression in a rat model of irritable bowel syndrome. Benef. Microbes.

[32] Yang Y.-J., Sheu B.-S. (2012). Probiotics-Containing Yogurts Suppress Helicobacter pylori Load and Modify Immune Response and Intestinal Microbiota in the Helicobacter pylori-Infected Children. Helicobacter.

[33] Pradhan D., Mallappa R.H., Grover S. (2019). Comprehensive approaches for assessing the safety of probiotic bacteria. Food Control.

[34] Zuo F., Chen S., Marcotte H. (2020). Engineer probiotic bifidobacteria for food and biomedical applications - Current status and future prospective. Biotechnol. Adv..

[35] FAO (2002). Guidelines for the Evaluation of Probiotics in Food.

[36] Li T., Teng D., Mao R., Hao Y., Wang X., Wang J. (2020). A critical review of antibiotic resistance in probiotic bacteria. Food Res. Int..

[37] Cui Y., Wang S., Ding S., Shen J., Zhu K. (2019). Toxins and mobile antimicrobial resistance genes in Bacillus probiotics constitute a potential risk for One Health. J. Hazard. Mater..

[38] Sanders M.E., Merenstein D.J., Ouwehand A., Reid G., Salminen S., Cabana M.D., Paraskevakos G., Leyer G. (2016). Probiotic use in at-risk populations. J. Am. Pharm. Assoc..

[39] Sotoudegan F., Daniali M., Hassani S., Nikfar S., Abdollahi M. (2019). Reappraisal of probiotics’ safety in human. Food Chem. Toxicol..

[40] Kirk M.D., Angulo F.J., Havelaar A.H., Black R.E. (2017). Diarrhoeal disease in children due to contaminated food. Bull. World Health Organ..

[41] Fung F., Wang H.-S., Menon S. (2018). Food safety in the 21st century. Biomed. J..

[42] Średnicka P., Juszczuk-Kubiak E., Wójcicki M., Akimowicz M., Roszko M. (2021). Probiotics as a biological detoxification tool of food chemical contamination: A review. Food Chem. Toxicol..

[43] Yang Y.-F., Chen C.-Y., Lu T.-H., Liao C.-M. (2018). Toxicity-based toxicokinetic/toxicodynamic assessment for bioaccumulation of polystyrene microplastics in mice. J. Hazard. Mater..

[44] Yao Y., Long M. (2020). The biological detoxification of deoxynivalenol: A review. Food Chem. Toxicol..

[45] Madreseh S., Ghaisari H.R., Hosseinzadeh S. (2018). Effect of Lyophilized, Encapsulated Lactobacillus fermentum and Lactulose Feeding on Growth Performance, Heavy Metals, and Trace Element Residues in Rainbow Trout (Oncorhynchus mykiss) Tissues. Probiotics Antimicrob. Proteins.

[46] Majlesi M., Shekarforoush S.S., Ghaisari H.R., Nazifi S., Sajedianfard J., Eskandari M.H. (2017). Effect of Probiotic Bacillus Coagulans and Lactobacillus Plantarum on Alleviation of Mercury Toxicity in Rat. Probiotics Antimicrob. Proteins.

[47] Yi Y.-J., Lim J.-M., Gu S., Lee W.-K., Oh E. (2017). Potential use of lactic acid bacteria Leuconostoc mesenteroides as a probiotic for the removal of Pb(II) toxicity. J. Microbiol..

[48] Kumar N., Kumar V., Panwar R., Ram C. (2016). Efficacy of indigenous probiotic Lactobacillus strains to reduce cadmium bioaccessibility - An in vitro digestion model. Environ. Sci. Pollut. Res..

[49] Khorshidian N., Yousefi M., Shadnoush M., Siadat S.D., Mohammadi M., Mortazavian A.M. (2020). Using probiotics for mitigation of acrylamide in food products: A mini review. Curr. Opin. Food Sci..

[50] Arribas-Lorenzo G., Morales F.J., Fishbein J.C. (2012). Chapter Five—Recent Insights in Acrylamide as Carcinogen in Foodstuffs. Advances in Molecular Toxicology.

[51] Bartkiene E., Jakobsone I., Juodeikiene G., Vidmantiene D., Pugajeva I., Bartkevics V. (2013). Study on the reduction of acrylamide in mixed rye bread by fermentation with bacteriocin-like inhibitory substances producing lactic acid bacteria in combination with Aspergillus niger glucoamylase. Food Control.

[52] Cuevas-González P., Aguilar-Toalá J., García H., González-Córdova A., Vallejo-Cordoba B., Hernández-Mendoza A. (2020). Protective Effect of the Intracellular Content from Potential Probiotic Bacteria against Oxidative Damage Induced by Acrylamide in Human Erythrocytes. Probiotics Antimicrob. Proteins.

[53] Fu L., Ning Y., Zhao H., Fan J., Zhang B. (2021). The In Vitro Adsorption Ability of Lactobacillus acidophilus NCFM to Benzo(a)pyrene in PM2.5. J. Toxicol..

[54] Shoukat S. (2020). Potential anti-carcinogenic effect of probiotic and lactic acid bacteria in detoxification of benzo[a]pyrene: A review. Trends Food Sci. Technol..

[55] Shoukat S., Liu Y., Rehman A., Zhang B. (2019). Screening of Bifidobacterium strains with assignment of functional groups to bind with benzo[a]pyrene under food stress factors. J. Chromatogr. B.

[56] Inturri R., Trovato L., Volti G.L., Oliveri S., Blandino G. (2019). In vitro inhibitory activity of Bifidobacterium longum BB536 and Lactobacillus rhamnosus HN001 alone or in combination against bacterial and Candida reference strains and clinical isolates. Heliyon.

[57] Ben Taheur F., Mansour C., Kouidhi B., Chaieb K. (2019). Use of lactic acid bacteria for the inhibition of Aspergillus flavus and Aspergillus carbonarius growth and mycotoxin production. Toxicon.

[58] Kosgey J.C., Jia L., Fang Y., Yang J., Gao L., Wang J., Nyamao R., Cheteu M., Tong D., Wekesa V. (2019). Probiotics as antifungal agents: Experimental confirmation and future prospects. J. Microbiol. Methods.

[59] Liu F., Malaphan W., Xing F., Yu B. (2019). Biodetoxification of fungal mycotoxins zearalenone by engineered probiotic bacterium Lactobacillus reuteri with surface-displayed lactonohydrolase. Appl. Microbiol. Biotechnol..

[60] Omotayo O.P., Omotayo A.O., Mwanza M., Babalola O.O. (2019). Prevalence of Mycotoxins and Their Consequences on Human Health. Toxicol. Res..

[61] Luo Y., Liu X., Yuan L., Li J. (2019). Complicated interactions between bio-adsorbents and mycotoxins during mycotoxin adsorption: Current research and future prospects. Trends Food Sci. Technol..

[62] Alu’Datt M.H., Rababah T., Sakandar H.A., Imran M., Mustafa N., Alhamad M.N., Mhaidat N., Kubow S., Tranchant C., Al-Tawaha A.R. (2018). Fermented food-derived bioactive compounds with anticarcinogenic properties: Fermented royal jelly as a novel source for compounds with health benefits. Anticancer Plants: Properties and Application.

[63] Kim S., Lee H., Lee S., Lee J., Ha J., Choi Y., Yoon Y., Choi K.-H. (2017). Invited review: Microbe-mediated aflatoxin decontamination of dairy products and feeds. J. Dairy Sci..

[64] Wochner K.F., Moreira M.C.C., Kalschne D.L., Colla E., Drunkler D.A. (2019). Detoxification of Aflatoxin B 1 and M 1 by Lactobacillus acidophilus and prebiotics in whole cow’s milk. J. Food Saf..

[65] Tajik H., Sayadi M. (2020). Effects of probiotic bacteria of *Lactobacillus acidophilus* and *Lactobacillus casei* on aflatoxin B_1_ detoxification within a simulated gastrointestinal tract model. Toxin Rev..

[66] Wu J.-C., Tsai M.-L., Lai C.-S., Lo C.-Y., Ho C.-T., Wang Y.-J., Pan M.-H. (2017). Polymethoxyflavones prevent benzo[a ]pyrene/dextran sodium sulfate-induced colorectal carcinogenesis through modulating xenobiotic metabolism and ameliorate autophagic defect in ICR mice. Int. J. Cancer.

[67] Halttunen T., Finell M., Salminen S. (2007). Arsenic removal by native and chemically modified lactic acid bacteria. Int. J. Food Microbiol..

[68] Zhai Q., Tian F., Zhao J., Zhang H., Narbad A., Chen W. (2016). Oral Administration of Probiotics Inhibits Absorption of the Heavy Metal Cadmium by Protecting the Intestinal Barrier. Appl. Environ. Microbiol..

[69] Clemente M.J., Vivó M.D.L., Puig S., Zúñiga M., Monedero V., Devesa V., Vélez D. (2020). In vitro evaluation of the efficacy of lactobacilli and yeasts in reducing bioavailability of inorganic arsenic. LWT.

[70] Hamad G.M., Taha T.H., E Hafez E., Ali S.H., A El Sohaimy S. (2017). Supplementation of Cerelac baby food with yeast–probiotic cocktail strains induces high potential for aflatoxin detoxification both in vitro and in vivo in mother and baby albino rats. J. Sci. Food Agric..

[71] Li B., Jin D., Yu S., Evivie S.E., Muhammad Z., Huo G., Liu F. (2017). In Vitro and In vivo Evaluation of Lactobacillus delbrueckii subsp. bulgaricus KLDS1.0207 for the Alleviative Effect on Lead Toxicity. Nutrients.

[72] Esfahani B.N., Kadivar M., Shahedi M., Soleimanian-Zad S. (2017). Reduction of acrylamide in whole-wheat bread by combining lactobacilli and yeast fermentation. Food Addit. Contam. Part A.

[73] Śliżewska K., Cukrowska B., Smulikowska S., Cielecka-Kuszyk J. (2019). The Effect of Probiotic Supplementation on Performance and the Histopathological Changes in Liver and Kidneys in Broiler Chickens Fed Diets with Aflatoxin B1. Toxins.

[74] Martínez B., Rodríguez A., Kulakauskas S., Chapot-Chartier M.-P. (2020). Cell wall homeostasis in lactic acid bacteria: Threats and defences. FEMS Microbiol. Rev..

[75] Chalova V.I., Lingbeck J.M., Kwon Y.M., Ricke S.C. (2008). Extracellular antimutagenic activities of selected probiotic Bifidobacterium and Lactobacillus spp. as a function of growth phase. J. Environ. Sci. Health Part B.

[76] He X., Zhao L., Zhang B., Zhao H. (2016). [Removal of benzo(a)pyrene by Lactobacillus strains under simulated starch conditions]. Acta Microbiol. Sin..

[77] Zhao H., Zhou F., Qi Y., Dziugan P., Bai F., Walczak P., Zhang B. (2013). Screening of Lactobacillus strains for their ability to bind Benzo(a)pyrene and the mechanism of the process. Food Chem. Toxicol..

[78] Arab M., Sohrabvandi S., Mortazavian A.M., Mohammadi R., Rezaei-Tavirani M. (2012). Reduction of aflatoxin in fermented milks during production and storage. Toxin Rev..

[79] Sarlak Z., Rouhi M., Mohammadi R., Khaksar R., Mortazavian A.M., Sohrabvandi S., Garavand F. (2017). Probiotic biological strategies to decontaminate aflatoxin M1 in a traditional Iranian fermented milk drink (Doogh). Food Control.

[80] Bahmani S., Azarpira N., Moazamian E. (2019). Anti-colon cancer activity of Bifidobacterium metabolites on colon cancer cell line SW742. Turk. J. Gastroenterol..

[81] Nowak A., Paliwoda A., Błasiak J. (2019). Anti-proliferative, pro-apoptotic and anti-oxidative activity of Lactobacillus and Bifidobacterium strains: A review of mechanisms and therapeutic perspectives. Crit. Rev. Food Sci. Nutr..

[82] Pei-Ren L., Chuiyu R., Cheng-Chun C., Ya-Hui T. (2002). Antimutagenic activity of several probiotic bifidobacteria against Benzo[a]pyrene. J. Biosci. Bioeng..

[83] Xu M., Fu L., Zhang J., Wang T., Fan J., Zhu B., Dziugan P., Zhang B., Zhao H. (2020). Potential of Inactivated Bifidobacterium Strain in Attenuating Benzo(A)Pyrene Exposure-Induced Damage in Colon Epithelial Cells In Vitro. Toxics.

[84] Valdez R.M.A., Ximenez-Fyvie L.A., Caiaffa K.S., dos Santos V.R., Cervantes R.M.G., Almaguer-Flores A., Duque C. (2020). Antagonist effect of probiotic bifidobacteria on biofilms of pathogens associated with periodontal disease. Microb. Pathog..

[85] Lili Z., Junyan W., Hongfei Z., Baoqing Z., Bolin Z. (2017). Detoxification of cancerogenic compounds by lactic acid bacteria strains. Crit. Rev. Food Sci. Nutr..

[86] Cecchini F., Morassut M., Saiz J.-C., Garcia-Moruno E. (2018). Anthocyanins enhance yeast’s adsorption of Ochratoxin A during the alcoholic fermentation. Eur. Food Res. Technol..

[87] Zhang Z., Li M., Wu C., Peng B. (2019). Physical adsorption of patulin by *Saccharomyces cerevisiae* during fermentation. J. Food Sci. Technol..

[88] Martínez M., Magnoli A., Pereyra M.G., Cavaglieri L. (2019). Probiotic bacteria and yeasts adsorb aflatoxin M1 in milk and degrade it to less toxic AFM1-metabolites. Toxicon.

[89] Jakopović Ž., Čiča K.H., Mrvčić J., Pucić I., Čanak I., Frece J., Pleadin J., Stanzer D., Zjalic S., Markov K. (2018). Properties and Fermentation Activity of Industrial Yeasts *Saccharomyces cerevisiae*, *S. uvarum*, *Candida utilis* and *Kluyveromyces marxianus* Exposed to AFB1, OTA and ZEA. Food Technol. Biotechnol..

[90] Luo Y., Wang J., Liu B., Wang Z., Yuan Y., Yue T. (2015). Effect of Yeast Cell Morphology, Cell Wall Physical Structure and Chemical Composition on Patulin Adsorption. PLoS ONE.

[91] Sahebghalam H., Sani A., Mehraban M. (2013). Bio-detoxification of AFB1 in animal feeds using *Saccharomyces cerevisiae*. J. Innov. Food Sci. Technol..

[92] Di Francesco A., Mari M., Ugolini L., Parisi B., Genovese J., Lazzeri L., Baraldi E. (2018). Reduction of acrylamide formation in fried potato chips by *Aureobasidum pullulans* L1 strain. Int. J. Food Microbiol..

[93] Waghmare S.R., Randive S.A., Jadhav D.B., Nadaf N.H., Parulekar R.S., Sonawane K.D. (2019). Production of novel antimicrobial protein from *Bacillus licheniformis* strain JS and its application against antibiotic-resistant pathogens. J. Proteins Proteom..

[94] Jin P., Tan Z., Wang H., Liu W., Miao W. (2020). Antimicrobial effect of Bacillus licheniformis HN-5 bacitracin A on rice pathogen *Pantoea ananatis*. BioControl.

[95] Yi P.-J., Pai C.-K., Liu J.-R. (2010). Isolation and characterization of a *Bacillus licheniformis* strain capable of degrading zearalenone. World J. Microbiol. Biotechnol..

[96] Yang Y., Pei J. (2020). Isolation and characterization of an Enterococcus strain from Chinese sauerkraut with potential for lead removal. Eur. Food Res. Technol..

[97] Mohsin M., Guenther S., Schierack P., Tedin K., Wieler L.H. (2015). Probiotic Escherichia coli Nissle 1917 reduces growth, Shiga toxin expression, release and thus cytotoxicity of enterohemorrhagic Escherichia coli. Int. J. Med Microbiol..

[98] Raghuvanshi R., Chaudhari A., Kumar G.N. (2017). 2-Ketogluconic acid and pyrroloquinoline quinone secreting probiotic Escherichia coli Nissle 1917 as a dietary strategy against heavy metal induced damage in rats. J. Funct. Foods.

[99] Nandy A., Basak S.C. (2000). Simple numerical descriptor for quantifying effect of toxic substances on DNA sequences. J. Chem. Inf. Comput. Sci..

[100] Weisburger J.H., Barnes W.S., Czerniak R. (2018). Mutagens and carcinogens in food. Diet, Nutrition, and Cancer: A Critical Evaluation.

[101] Križková L., Belicová A., Dobias J., Krajčovič J., Ebringer L. (2002). Selenium enhances the antimutagenic activity of probiotic bacterium Enterococcus faecium M-74. World J. Microbiol. Biotechnol..

[102] Lim S.-M. (2013). Antimutagenicity activity of the putative probiotic strain Lactobacillus paracasei subsp. tolerans JG22 isolated from pepper leaves Jangajji. Food Sci. Biotechnol..

[103] Sah B., Vasiljevic T., McKechnie S., Donkor O. (2015). Effect of refrigerated storage on probiotic viability and the production and stability of antimutagenic and antioxidant peptides in yogurt supplemented with pineapple peel. J. Dairy Sci..

[104] Chandel D., Sharma M., Chawla V., Sachdeva N., Shukla G. (2019). Isolation, characterization and identification of antigenotoxic and anticancerous indigenous probiotics and their prophylactic potential in experimental colon carcinogenesis. Sci. Rep..

[105] Sah B.N.P., Vasiljevic T., McKechnie S., Donkor O.N. (2015). Effect of pineapple waste powder on probiotic growth, antioxidant and antimutagenic activities of yogurt. J. Food Sci. Technol..

[106] Song M., Yun B., Moon J.-H., Park D.-J., Lim K., Oh S. (2015). Characterization of Selected Lactobacillus Strains for Use as Probiotics. Korean J. Food Sci. Anim. Resour..

[107] Ritchie H., Roser M. (2018). Causes of Death. Our World in Data. https://ourworldindata.org/causes-of-death.

[108] Shamekhi S., Abdolalizadeh J., Ostadrahimi A., Mohammadi S.A., Barzegari A., Lotfi H., Bonabi E., Zarghami N. (2020). Apoptotic Effect of *Saccharomyces cerevisiae* on human colon cancer SW480 cells by regulation of Akt/NF-ĸB signaling pathway. Probiotics Antimicrob. Proteins.

[109] Hibberd A.A., Lyra A., Ouwehand A.C., Rolny P., Lindegren H., Cedgård L., Wettergren Y. (2017). Intestinal microbiota is altered in patients with colon cancer and modified by probiotic intervention. BMJ Open Gastroenterol..

[110] Zhang T., Pan D., Yang Y., Jiang X., Zhang J., Zeng X., Wu Z., Sun Y., Guo Y. (2020). Effect of *Lactobacillus acidophilus* CICC 6074 S-Layer Protein on Colon Cancer HT-29 Cell Proliferation and Apoptosis. J. Agric. Food Chem..

[111] Motevaseli E., Dianatpour A., Ghafouri-Fard S. (2017). The Role of Probiotics in Cancer Treatment: Emphasis on their In vivo and In Vitro Anti-metastatic Effects. Int. J. Mol. Cell. Med..

[112] Panebianco C., Latiano T., Pazienza V. (2020). Microbiota Manipulation by Probiotics Administration as Emerging Tool in Cancer Prevention and Therapy. Front. Oncol..

[113] Śliżewska K., Markowiak-Kopeć P., Śliżewska W. (2020). The Role of Probiotics in Cancer Prevention. Cancers.

[114] Pescuma M., Gomez-Gomez B., Perez-Corona T., Font G., Madrid Y., Mozzi F. (2017). Food prospects of selenium enriched-Lactobacillus acidophilus CRL 636 and Lactobacillus reuteri CRL 1101. J. Funct. Foods.

[115] Rajoka M.S.R., Zhao H., Lu Y., Lian Z., Li N., Hussain N., Shao D., Jin M., Li Q., Shi J. (2018). Anticancer potential against cervix cancer (HeLa) cell line of probiotic *Lactobacillus casei* and *Lactobacillus paracasei* strains isolated from human breast milk. Food Funct..

[116] Utz V.E.M., Visñuk D.P., Perdigón G., Leblanc A.d.M.d. (2020). Milk fermented by Lactobacillus casei CRL431 administered as an immune adjuvant in models of breast cancer and metastasis under chemotherapy. Appl. Microbiol. Biotechnol..

[117] Silveira D.S.C., Veronez L.C., Lopes-Júnior L.C., Anatriello E., Brunaldi M.O., Pereira-Da-Silva G. (2020). *Lactobacillus bulgaricus* inhibits colitis-associated cancer via a negative regulation of intestinal inflammation in azoxymethane/dextran sodium sulfate model. World J. Gastroenterol..

[118] Kahouli I., Malhotra M., Westfall S., Alaoui-Jamali M.A., Prakash S. (2016). Design and validation of an orally administrated active L. fermentum-L. acidophilus probiotic formulation using colorectal cancer Apc Min/+ mouse model. Appl. Microbiol. Biotechnol..

[119] Ghidini M., Nicoletti M., Ratti M., Tomasello G., Lonati V., Ghilardi M., Parati M., Borgonovo K., Cabiddu M., Petrelli F. (2021). Lactobacillus Kefiri LKF01 (Kefibios^®^) for Prevention of Diarrhoea in Cancer Patients Treated with Chemotherapy: A Prospective Study. Nutrients.

[120] Xiao L., Ge X., Yang L., Chen X., Xu Q., Rui X., Fan X., Feng L., Zhang Q., Dong M. (2020). Anticancer potential of an exopolysaccharide from *Lactobacillus helveticus* MB2-1 on human colon cancer HT-29 cells *via* apoptosis induction. Food Funct..

[121] Luo M., Hu M., Feng X., XiaoLi W., Dong D., Wang W. (2020). Preventive effect of Lactobacillus reuteri on melanoma. Biomed. Pharmacother..

[122] Rahimi A.M., Nabavizadeh F., Ashabi G., Halimi S., Rahimpour M., Vahedian J., Panahi M. (2020). Probiotic Lactobacillus rhamnosus Supplementation Improved Capecitabine Protective Effect against Gastric Cancer Growth in Male BALB/c Mice. Nutr. Cancer.

[123] Ohara T., Suzutani T. (2018). Intake of Bifidobacterium longum and Fructo-oligosaccharides prevents Colorectal Carcinogenesis. Euroasian J. Hepato-Gastroenterol..

[124] Jaskulski I.B., Uecker J., Bordini F., Moura F., Gonçalves T., Chaves N.G., Camargo F., Grecco F.B., Fiorentini M., da Silva W.P. (2019). In vivo action of Lactococcus lactis subsp. lactis isolate (R7) with probiotic potential in the stabilization of cancer cells in the colorectal epithelium. Process Biochem..

